# The Extracellular Matrix and Remyelination Strategies in Multiple Sclerosis

**DOI:** 10.1523/ENEURO.0435-17.2018

**Published:** 2018-04-06

**Authors:** Yuyi You, Vivek Gupta

**Affiliations:** 1 Save Sight Institute, Sydney Medical School, The University of Sydney, NSW 2000, Sydney, Australia; 2 Faculty of Medicine and Health Sciences, Macquarie University, NSW 2109, Sydney, Australia

**Keywords:** demyelination, extracellular matrix, multiple sclerosis, remyelination

## Significance Statement

Remyelination therapy for multiple sclerosis (MS) is a rapidly emerging research area despite the fact that only limited success has been achieved so far in clinical trials. The extracellular matrix (ECM) is significantly altered in chronic MS lesions, which is believed to be an important remyelination-inhibiting factor. However, the ECM components have not been specifically targeted in current MS remyelinating trials. Qin et al. (2017) described the role of a major ECM protein, fibronectin, in de/remyelination. Exogenous ganglioside GD1a was demonstrated to overcome the remyelination-inhibiting effects of aggregated fibronectin during later stages of oligodendrocyte maturation. Thus, GD1a could potentially be used as a novel remyelinating compound or as combination therapy in conjunction with other drugs to enhance different stages of remyelination in MS.

## 

Multiple sclerosis (MS) is an autoimmune disorder of the central nervous system, characterized by inflammatory demyelination and progressive axonal loss. Demyelination has long been considered as the main pathologic feature of MS. Remyelination usually fails in chronic MS lesions despite the presence of oligodendrocyte precursor cells (OPCs; Kuhlmann et al., 2008). A major focus of current MS treatment is on immunomodulation and relapse control. However, it is increasingly believed that chronic demyelination can cause secondary axonal loss (Nave, 2010), which may lead to disease progression and clinical disability. Therefore, remyelinating therapy has become a rapidly emerging MS research area (Plemel et al., 2017). Some novel remyelinating compounds have been developed and progressed into clinical trials. Most of the remyelination clinical trials included patients with optic neuritis as study subjects, because de/remyelination in the visual pathways is more clinically measureable, which can be determined by the latency of visual evoked potentials (VEPs; You et al., 2011). However, only limited success in VEP latency improvement has been achieved to date in the clinical trials (Cadavid et al., 2017; Green et al., 2017). Considering the fact that multiple proteins and signaling pathways are involved in the myelin pathology of the disease, targeting only one pathway might not be enough to generate substantial remyelination in MS lesions.

**Figure 1.**
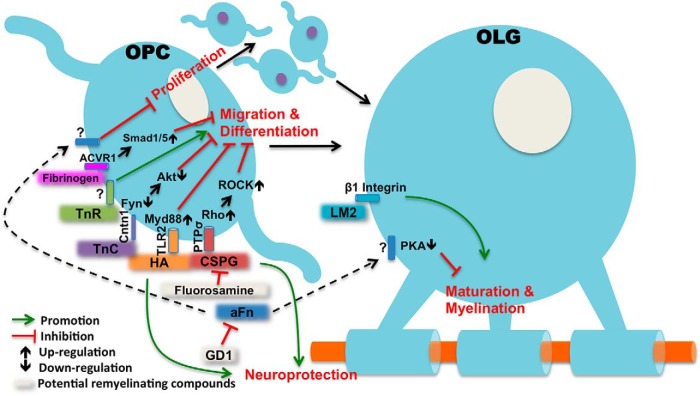
The role of ECM components in oligodendrocyte (OLG) remyelination. Multiple ECM components are inhibitors of OPC differentiation and migration, including CSPGs; (Lau et al., 2013; Pendleton et al., 2013), hyaluronan (HA; Sloane et al., 2010), TnC (Czopka et al., 2010), and fibrinogen (Petersen et al., 2017) via different signaling pathways. Laminin-2 (LM2) may not have significant effects on OPC differentiation, but it promotes myelin sheets formation in mature OLGs (Buttery and ffrench-Constant, 1999). TnR is also a pro-remyelinating component, and it improves OPC adhesion and differentiation (Pesheva et al., 1997). Aggregated fibronectin (aFn) in the highlighted paper is an inhibitor of OPC proliferation and OLG myelin formation (Qin et al., 2017), although the exact mechanisms are still unclear. Administration of GD1a (Qin et al., 2017), fluorasamine (Keough et al., 2016), and ancrod (Petersen et al., 2017) in animals can overcome the remyelination-inhibiting effects of aFn, CSPG, and fibrinogen, respectively. Thus, those compounds have translational potentials for remyelinating therapy. ACVR1, activin A receptor, Type I; PTPσ, protein tyrosine phosphatase sigma; ROCK, Rho-associated protein kinase.

The extracellular environment, or extracellular matrix (ECM), is significantly altered inside MS plaques (van Horssen et al., 2007), which is considered to be an important remyelination-inhibiting factor in chronic lesions. However, the ECM has not been targeted in the current MS remyelinating trials, which could potentially explain the relatively unsuccessful clinical results so far. This may be particularly true for chronic lesions, where some ECM components can form a non-permissive barrier at the lesion edge to block migration of OPCs to the lesion core, leading to a reduced OPC population with impaired potential for remyelination and lesion repair (Lau et al., 2013). The ECM is composed of proteoglycans, hyaluronan and multiple protein components such as collagen, fibronectin and laminin. Recent studies have suggested that most of the major ECM components may play a role in OPC migration and differentiation. Chondroitin sulfate proteoglycans (CSPGs) were shown to accumulate in demyelinating lesions and have multiple inhibitory actions on oligodendrocytes (Pendleton et al., 2013; Keough et al., 2016). The glycosaminoglycan hyaluronan was also identified in MS lesions (Back et al., 2005) and was found to be an inhibitor of OPC maturation and remyelination through Toll-like receptor 2 (TLR2; Sloane et al., 2010). Type IV collagen is an important basement membrane protein, and an increased collagen deposit has been seen in MS lesions which was thought to be an inhibitor of OPC migration (van Horssen et al., 2007). In contrast, laminin-2 can significantly enhance myelin membrane formation and promote remyelination (Buttery and ffrench-Constant, 1999). On the other hand, tenascins, including tenascin-C (TnC) and tenascin-R (TnR), appear to play opposite roles in remyelination. TnC was shown to inhibit OPC differentiation via cell adhesion molecule contactin (Cntn1; Czopka et al., 2010), while TnR can potentially promote OPC adhesion and differentiation (Pesheva et al., 1997). In addition, fibrinogen, the plasma protein, can also enter the brain parenchyma and inhibit OPC differentiation when there is disruption of the blood brain barrier under certain disease conditions including MS (Baeten and Akassoglou, 2011; Petersen et al., 2017). The major ECM components and their roles in OPC differentiation and remyelination are summarized in Figure 1. The ECM in MS lesions forms a complex network of interacting proteoglycans and proteins and the predominant signaling pathway remains to be determined (Plemel et al., 2017).

The recent paper (Qin et al., 2017) published in the *Journal of Neuroscience* has broadened our understanding of the role of fibronectin (Fn), another major ECM component, in MS de/remyelination. This study was developed based on the group’s previous investigations (Stoffels et al., 2013), where they demonstrated Fn aggregation in MS lesions as well as in experimental autoimmune encephalitis (EAE) but not in the lysolecthin-induced demyelination model. The results suggested that Fn aggregation is mediated by inflammatory demyelination and inhibits oligodendrocyte remyelination. The authors further investigated the underlying mechanisms as well as the therapeutic potential of overcoming Fn-mediated deficits of myelin formation. It was first shown in OPC cultures that gangliosides GD1a corrected the inhibition of myelin membrane formation induced by aggregated Fn. This remyelination effect appears to be generated via OPC proliferation and myelin formation rather than cell migration or differentiation. The results were consistent with the findings in a previous study where OPCs were cultured on Fn-coated dishes and no effect on cell differentiation was observed (Baron et al., 2014). By contrast, it was suggested by other studies that failure of remyelination in chronic MS lesions might be attributed primarily to reduced OPC recruitment (Boyd et al., 2013) or differentiation (Kuhlmann et al., 2008). Also, the effect of Fn on OPC migration remains to be determined. It was not surprising to see an increased level of myelin proteolipid protein (PLP) mRNA *in vivo* after GD1a treatment. This again is likely to be a result of OPC proliferation, evidenced by increased Ki67(+) OPCs. In the *in vivo* study, the authors demonstrated an unchanged percentage of MBP-positive cells, suggesting no effects on OPC differentiation; therefore, a similar percentagewise analysis (e.g., ratio of myelin producing oligodendrocytes to total oligodendrocyte lineage “Olig2” cells) could potentially reveal whether GD1a affects OPC differentiation *in vivo*. Additionally, it remains to be confirmed whether the PLP mRNA upregulation could eventually lead to an enhanced myelin sheath formation. It is challenging to test this *in vivo*, Fn aggregation is only seen in the EAE model (not in the cuprizone or lysolecthin models), but the EAE model is not ideal for remyelination study because of ongoing inflammation and sporadic demyelinating lesions with unpredictable lesional site and timing; therefore, exogenous aggregated Fn had to be added in the cuprizone model in the highlighted study. Spontaneous aggregate clearance over time leads to only a transient aggregated Fn microenvironment *in vivo*, which makes it difficult to study ultimate remyelination. It may, therefore, be worthwhile to consider inducing EAE in a transgenic mouse line, which allows OPC labeling and lineage analysis (Mei et al., 2016).

Next, Qin et al. (2017) investigated the mechanisms of GD1a-induced remyelination. It was demonstrated that only in the late stage of oligodendrocyte maturation does GD1a become effective in promoting myelin membrane formation. By using the serine/threonine kinase (STK) array, the authors identified that the above GD1a effects on myelin formation were mediated through the activation of the protein kinase A (PKA)-signaling pathway, which was further confirmed in oligodendrocyte cultures by analyzing the PKA downstream cAMP response element-binding protein (CREB) as well as by using the PKA inhibitor H89 and activator dBcAMP. While the effect might be mediated via oligodendrocyte membrane microdomains as suggested by the authors, the detailed machinery of GD1a-induced PKA activation remains to be determined. Also, how GD1a is inducing OPC proliferation at the early stage of remyelination is still unknown. Nevertheless, the results from this paper have significantly extended our knowledge of the ECM-related signaling pathways in MS remyelination (Fig. 1).

As shown in Figure 1, since the ECM constitutes a complex signaling network in OPC differentiation and myelin formation, targeting only one ECM component might not be sufficient for successful remyelination in real disease scenarios. There are some major obstacles to overcome in developing remyelination therapies for MS. Firstly, OPC recruitment and differentiation is believed to play a key role in the process of successful remyelination and therefore most of the new potential remyelinating drugs are focusing on targeting OPC differentiation. Interestingly, GD1a was not shown to have significant effects on OPC differentiation. This provides a possibility that GD1a can potentially be used in combination with other drugs to form double or even triple therapies to enhance different stages of remyelination. Secondly, a pathologic feature of MS lesions is astrogliotic scarring. Most of the ECM-based remyelination inhibitors are astrocyte driven, including CSPGs, hyaluronan, as well as aggregated Fn (Stoffels et al., 2013). Astrocytes play a complex dual role in remyelination. It has been well documented that astrocytic signaling is required for OPC survival and differentiation (Moore et al., 2011), but on the other side, reactive astrocytes shown in human MS are not only neurotoxic, but also toxic to differentiated oligodendrocytes (Liddelow et al., 2017). Finally, it has been recognized that there is primary neurodegeneration in MS, evidenced by progressive retinal nerve fiber loss in non-optic neuritis eyes (Graham et al., 2016; Petzold et al., 2017), as well as by morphologic (Petzold et al., 2017) and functional (You et al., 2018) changes in the myelin deficient retinal inner nuclear layer. Therefore, it may be essential to incorporate neuroprotection as part of the therapeutic strategy to ensure successful remyelination. Interestingly, some of the above remyelination-inhibiting ECM components (e.g., CSPGs, hyaluronan) are important in maintaining synaptic plasticity and were found to be neuroprotective in the central nervous system (Suttkus et al., 2016).

In summary, the ECM comprises an extremely complex neural signaling network, with both pro- and counter-remyelinating components coexisting, and myelin-formation inhibitors being neuroprotectants. The predominant cellular signaling pathway mediating remyelination in MS is not well understood. However, recent advances in pre-clinical models have significantly improved our knowledge about the role of ECM in MS pathology, bringing us steps closer to its potential clinical applications.

## Synthesis

Reviewing Editor: Jun Chen, University of Pittsburgh Decisions are customarily a result of the Reviewing Editor and the peer reviewers coming together and discussing their recommendations until a consensus is reached. When revisions are invited, a fact-based synthesis statement explaining their decision and outlining what is needed to prepare a revision will be listed below. The following reviewer(s) agreed to reveal their identity: Stephen Crocker.

In general, the manuscript is well written and the topic timely. However, there are rooms for improvement, especially the comments from Reviewer #2 are more substantial and constructive.

Reviewer #1

There are some points in the writing, which is generally quite good, that could benefit from clarification that the author(s) may consider:

1. Sentence staring on line 65 (“It was not...”) is quite labored in its use of commas and could be conveyed with two sentences instead of its current form.

2. line 77: another drawback that author(s) could consider adding to the mention of the EAE model for study of remyelination is that the myelin lesions in these models are sporadic and unpredictable in anatomical site and timing.

3. Line 82: the citation being placed in the middle of the sentence is unusual and given that the statement following the comma after that reference is a continuation of the primary point of that sentence the reference would be best served being placed at the end of the sentence.

4. line 88: please define STK array or provide a more specific statement about the methodology used in the study.

5. line 138: The figure caption could benefit from a narrative introduction to this summary. References in the figure legend could also be ordered to specifically refer to each molecule as they are listed.

Reviewer #2

There are the following shortcomings with the article that need to be improved:

1) Emphasis on one recent study on fibronectin is not representative of the work in the field that has studied multiple ECM proteins in myelination and remyelination. Since the perspective is entitled “ECM and remyelination strategies in MS” it should present a balanced view of the ECM proteins in remyelination in MS.

2) A recently published study on the role of fibrinogen as an inhibitor of OPC maturation and remyelination should be cited and discussed (Petersen et al., 2017, Neuron 96, p1003-1012). Figure 1 should also be updated to include fibrinogen. The contribution of fibrinogen to ECM is well established (Baeten and Akassoglou 2011, Dev Neurobiol 71, p1018-39).

3) Given that multiple ECM components interfere with remyelination, thoughts on how to prioritize targeting these ECM proteins would be helpful to be included. Would targeting only one be enough?

4) The remyelinating compounds have not been tested yet in MS. The trials cited are in optic neuritis. The sentence in page 1 stating that the remyelinating compounds “have progressed into clinical trials but only limited success has been achieved” is misleading as it leaves the impression that these compounds have been tested in MS. It needs to be clarified that these are very early trials and MS trials are still ongoing.
